# Bioactivity In Vitro of Quercetin Glycoside Obtained in *Beauveria bassiana* Culture and Its Interaction with Liposome Membranes

**DOI:** 10.3390/molecules22091520

**Published:** 2017-09-11

**Authors:** Paulina Strugała, Tomasz Tronina, Ewa Huszcza, Janina Gabrielska

**Affiliations:** 1 Department of Physics and Biophysics, Wrocław University of Environmental and Life Sciences, Norwida 25, 50-375 Wrocław, Poland; janina.gabrielska@upwr.edu.pl; 2 Department of Chemistry, Wrocław University of Environmental and Life Sciences, Norwida 25, 50-375 Wrocław, Poland; tomasz.tronina@upwr.edu.pl (T.T.); ewa.huszcza@upwr.edu.pl (E.H.)

**Keywords:** quercetin glycoside, microbial glycosylation, liposomes, fluidity, antioxidants, COX-1, COX-2, human serum albumin

## Abstract

Quercetin (Q) was used as substrate for regioselective glycosylation at the C-7 position catalyzed by *Beauveria bassiana* AM278 strain. As a result the glycoside quercetin 7-*O*-β-d-(4″-*O*-methyl)glucopyranoside (Q 7-MeGlu) was formed. The goal of the studies was to determine the anti-oxidative (liposome membrane protection against free radicals IC_50_^Q 7-MeGlu^ = 5.47 and IC_50_^Q^ = 4.49 µM) and anti-inflammatory (COX-1 and COX-2 enzymes activity inhibition) properties of Q 7-MeGlu as compared to Q. Every attempt was made to clarify the antioxidant activity of these molecules, which are able to interact with egg phosphatidylcholine liposomes, using a fluorometric method (by applying the probes MC540, TMA-DPH and DPH). The results indicated that Q 7-MeGlu and Q are responsible for increasing the packing order, mainly in the hydrophilic but also in hydrophobic regions of the membrane (Q > Q 7-MeGlu). These observations, confirmed by a ^1^H-NMR method, are key to understanding their antioxidant activity which is probably caused by the stabilizing effect on the lipid membranes. The results showed that Q 7-MeGlu and Q have ability to quench the human serum albumin (HSA) intrinsic fluorescence through a static quenching mechanism. The results of thermodynamic parameters indicated that the process of formation complexes between studied molecules and HSA was spontaneous and caused through Van der Waals interactions and hydrogen bonding.

## 1. Introduction

Quercetin (3,3′,4′,5,7-pentahydroxyflavone), a major bioflavonoid found in fruits and vegetables, exhibits unique biological properties [1]. A broad spectrum of beneficial quercetin properties, including antimutagenic, antifibrotic, antiinflammatory, antiatherogenic, and antibacterial effects, as well as its strong antioxidative capacity, is described. In addition, the most relevant factors seem to be antihypertensive effects on human body and the improvement of endothelial function [2,3,4]. Quercetin is responsible for reducing viability and inducing apoptosis of numerous cancer cells lines, including those from breast, hepatic, colon, lung, ovary, endometrial cancers and prostate cancers. Quercetin can also control cancer cell growth by the regulation of specific signaling pathways, e.g., decreasing oncogene expression, inducing malignant cell apoptosis and inhibiting angiogenesis [5,6]. Due to its antiproliferative and proapoptotic properties quercetin is a promising, natural compound which can be used supportively in chemotherapy. Quercetin proves to be an excellent in vitro antioxidant. Considering the whole family of flavonids, it seems to be the most potent scavenger of reactive oxygen/nitrogen species, including superoxide anion radicals, hydroxyl radicals, peroxynitrite and peroxyl radicals. The presence of the *ortho*-dihydroxy substitution in the B-ring, 2,3-unsaturation, a 4-carbonyl in the C-ring as well as the OH group at position 3, which are responsible for these high antioxidative capacities of quercetin, is observed [3,7]. It is proved that the daily intake of this flavanol in the diet is estimated around 5–40 mg/day. However, it is possible to increase these levels up to 200–500 mg/day for human beings by consumption high quantities of fruits and vegetables e.g., onions, apple skin, tomatoes, lettuce, cauliflower, and celery, which are an important source of flavonols [8]. Due to its beneficial therapeutic potential, quercetin is likely to be used in therapy of many diseases. However, its poor bioavailability, low aqueous solubility, and its low circulation time in the body support the view that the application is still limited in use. Very important determinants which impact better water solubility of flavonoids is presence of hydroxyl groups as well as sugar moieties. It is well known fact that in the same conditions (temperature and pH value below 7) flavonoid glycosides are better soluble in water than their aglycones. The solubility of aglycone naringenin at 20 °C is 4.38 mg/L [9] whereas for its glycoside naringin it is 500 mg/L (more than 110- fold higher), [10]. The same trend is observed in case of rutin and quercetin. Solubility of rutin in water is 125 mg/L [11] whereas for quercetin aglycone it is 0.512 mg/L (more than 240-fold lower) [12]. Therefore glycosylation can be defined as a process of improving the solubility of flavonoids such as quercetin what is broadly accepted as an effective method for conversion of water-insoluble and unstable organic compounds into the corresponding water-soluble and chemically stable ones [13]. Quercetin without sugar groups was initially believed to be taken up in the gastro-intestinal tract by passive diffusion which was explained by the hydrophilic character of its glycosides. In another study, it was proved that quercetin glycosides exhibit higher bioavailability than the aglycone and simultaneously quercetin glucosides are absorbed more rapidly than other types of glycosides, e.g., those of rutin [3,14,15]. It was also shown that the type of attached sugar moiety had a great impact on absorption of quercetin in vivo. Quercetin glucosides were absorbed 10 times faster than rutinosides in humans [16]. Moreover, the absorption of the 3-*O*-glucoside of quercetin was 184% higher compared to quercetin itself in the rat model [17].

Interest towards biological approaches for glycosylating flavonoids and other compounds has increased greatly. Biotransformations are conducted under mild conditions and due to their high stereo- and regio-selectivity are an extremely useful tool for obtaining novel compounds, including ones which do not exist in Nature [18,19]. In addition, a significant therapeutic impact in comparison to the initial substrates is observed as a consequence of the fact that biotransformations are frequently the source of derivatives which show greater biological activity than the parent compound. Moreover, they provide an environmentally friendly alternative for fine chemical synthesis. In addition, according to the EU regulations (EU directive 88/388/EEC) all the products which are produced in the process of biotransformation of natural compounds are also classified as natural products. Biotransformations conducted by fungal cultures are the fundamental methods for obtaining glycosylated flavonoids from their aglycones. What is more, one of the key advantages of whole-cell biotransformation is the possibility to eliminate the need for isolating, purifying and stabilizing enzymes [20]. In particular, the application of fungal strains (*Beauveria bassiana* AM 278, *Absidia glauca* AM 177 and *Absidia coerulea* AM 93) for biotransformation of flavonoids (*inter alia* daidzein, genistein, xantohumol) led to formation of many useful novel compounds by means of glycosylation reactions [21,22,23,24].

Considering the biological properties of flavonoids, their ability to enter into chemical reactions with the lipid bilayer, which is the main and sometimes the only place of interaction between various physical and chemical factors on a living organism, is fundamental [25]. On the basis of experimental research it may be stated that natural medicines, which are able to change the membrane properties, can also generate multiple effects leading finally to *inter alia*: antioxidative and anticancer properties of the compounds. Thus, the ability of flavonoids to interact with the cell membrane is important from the medical and biological points of view and is, to some degree, connected with the use of these natural, antioxidative compounds as antioxidants and anticancer drugs. In case of newly discovered compounds from the group of flavonoids, e.g., via the process of biotransformation, it is appropriate to conduct complex studies of their biological properties in order to clarify the molecular mechanisms which are responsible for the protection of the lipid bilayer and which are against the peroxidation processes. In the present studies, liposomes from egg phosphatidylcholine (PC) were selected as a result of the fact that their composition is similar to the natural cell bilayer lipid membrane.

Due to its interesting biological activities, structural modification of quercetin was conducted using a fungal strain (*Beauveria bassiana* AM 278) to obtain a new derivative. The aim was to obtain a glycosylated derivative which shows promising biological properties by a quercetin microbiological transformation process. The broad spectrum of quercetin’s biological activity was crucial for conducting a wide range of biological studies on this chemical compound. The goal of the studies was to determine: (1) antioxidative activity in relation to model lipid membranes in which the process of peroxidation was induced by the chemical compound AAPH; (2) conduct complex research (fluorometric and ^1^H-NMR methods) on model lipid membranes in order to localize the site of interaction between the compounds and the lipid bilayer as well as the impact of those compounds on the membrane; (3) determine the potential anti-inflammatory activity in the enzymatic inhibition of pro-inflammatory enzymes using the COX-1 and COX-2 method; (4) analyze the binding mechanism between the compounds and the main blood protein—human albumin. To the best of our knowledge, the obtained quercetin derivative, i.e., quercetin 7-*O*-β-d-(4″-*O*-methyl)gluco-pyranoside, have not been biologically tested before.

## 2. Results and Discussion

### 2.1. Chemistry

#### Biotransformation Catalyzed by *Beuaveria bassiana* AM278

**Scheme 1:**
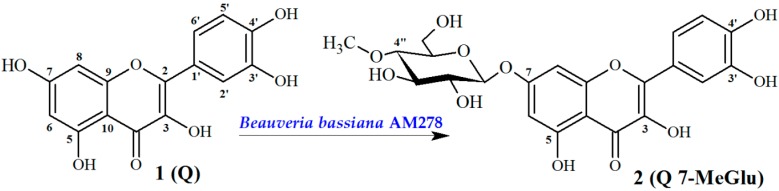
Regioselective glycosylation of quercetin (Q) catalyzed by *B. bassiana* AM278.

The fungus *Beauveria bassiana* is known for its ability to modify dozens of different chemical compounds, therefore it is among the most frequently used whole cell biocatalysts [26]. It is able to catalyze a wide range of reactions, including oxidation, reduction, hydroxylation, methylation as well as conjugation of polar molecules, e.g., sulphate ions or sugar molecules. Studies on biotransformations of numerous different flavonoids by *B. bassiana* have shown that the most common modification was the introduction of 4-*O*-methylglucopyranose [21,22,24,27]. The reaction was highly regioselective and the preferred position of glycosylation was the hydroxyl group at C7 in the A-ring of flavonoid molecules. As expected, the biotransformation of quercetin (Q) catalyzed by *B. bassiana* AM278 (Scheme 1) led to its glucoside-quercetin 7-*O*-β-d-(4″-*O*-methyl)glucopyranoside (Q 7-MeGlu). The structure of the obtained metabolite Q 7-MeGlu was determined by NMR and MS methods.

HR ESI-MS spectrum of metabolite Q 7-MeGlu showed the [M − H]^−^ peak at *m*/*z* 477.1037 (Supplementary Materials Figure S2) that was consistent with the molecular formula of C_22_H_22_O_12_ (calculated for C_22_H_22_O_12_ − H ([M − H]^−^ 477.1038). This proved that compared with the aglycone Q the metabolite Q 7-MeGlu contains seven additional carbon atoms. Several remarkable differences were observed in the ^1^H-NMR and ^13^C-NMR spectra of the metabolite Q 7-MeGlu compared to quercetin (Supplementary Materials, Figure S3 and S4). The presence of new ^1^H-NMR signals in the region ranging from δ_H_ 3.0–5.1 ppm and seven new signals in the δ_C_ 59.7–99.5 ppm range of the ^13^C-NMR spectrum of the compound Q 7-MeGlu (Supplementary Materials, Figure S4) suggested that a sugar moiety was conjugated to the quercetin. The values of chemical shift of additional signals proved that the attached sugar was glucopyranose. However, the chemical shift value of the C-4 signal (C-4″) of the glucose molecule was abnormal. This signal is normally present in the region ranging from δ_C_ 70.0–71.5 ppm. In the case of Q 7-MeGlu spectra, it was observed at a lower magnetic field at δ_C_ 78.8 ppm. This shift indicated that instead of glucose its C-4 (C4″)-substituted derivative was involved in glucosylation (Supplementary Materials, Figure S5). The seventh additional carbon atom present in ^13^C-NMR spectrum of Q 7-MeGlu appeared at δ_C_ 59.7 ppm and showed a correlation in the HSQC spectrum with a three proton singlet at δ_H_ 3.46 ppm, which confirmed the presence of a methoxyl group (Supplementary Materials, Figure S7). To sum up, the chemical shift values of the seven additional carbon atoms which appeared in ^13^C-NMR spectrum of Q 7-MeGlu were characteristic for the 4-*O*-methyl-glucopyranose moiety. This sugar is very often conjugated to substrate molecules in glycosylation reactions catalyzed by *B. bassiana* [22,24,27]. The correlations between protons and carbon atoms presented in the HSQC spectrum of the metabolite Q 7-MeGlu allowed us to assign accurate positions of each proton signal of the 4-*O*-methylglucopyranose molecule despite some of them being overlapped with each other (Supplementary Materials, Figure S7). The shifts of the H-6 and H-8 signals to a lower magnetic field (δ_H_ 6.19 → 6.42 ppm and δ_H_ 6.41 → 6.76 ppm, respectively) as well as that of the signal of C-7 to higher field (δ_C_ 162.6 → 163.7 ppm) proved without any doubts that the sugar moiety was conjugated to the hydroxyl group attached to C-7. Studies on the regioselectivity of glucosylation catalyzed by *B. bassiana* strains have indicated that this is a preferable position for sugar moiety addition to numerous natural and synthetic flavonoids, even if they have significant differences in structure (different skeletons) and presence and positions of hydoxyl groups [21,22,24,27,28]. The rest of the ^1^H- and ^13^C-NMR signals in the spectra of the metabolite Q 7-MeGlu were very similar to those of the substrate Q and suggested that the introduction of 4-*O*-methyl-glucopyranose moiety to ring A at position C-7-OH was the only reaction catalyzed by the tested *B. bassiana* AM278 strain. The NMR and MS data thus fully confirmed the structure of quercetin 7-*O*-β-d-(4″-*O*-methyl)glucopyranoside (Q 7-MeGlu) as a biotransformation product of quercetin (Q) catalyzed by *B. bassiana* AM278.

### 2.2. Interaction with Lipid Membrane

#### 2.2.1. Fluorescence Measurements—MC540, TMA-DPH and DPH Probes

By using the fluorometric probe MC540, TMA-DPH and DPH, which were localized at different depth of lipid bilayer membrane, the impact of Q 7-MeGlu and Q on the hydrophilic and hydrophobic properties of the regions in the model phospholipid membrane was specified. The changes in probe’s intensity/anisotropy (MC540, TMA-DPH, and DPH): in comparison with the control (without addition of studied compounds) caused by Q 7-MeGlu and Q in concentration 1, 5 and 10 µM are presented in Table 1.

Probe MC540 is an amphipathic anionic molecule and its negative charge determines its location at or near the membrane interface, slightly above the domain of the glycerol backbone of neutral and charged phospholipids. A study conducted by Alay et al. [29] showed that MC540 binding is very sensitive to the lipid packing of phospholipid bilayers. The results of our studies showed that Q 7-MeGlu at a concentration 1–10 μM caused a reduction in intensity of the MC540 probe’s fluorescence within the range from ca. 3 up to ca. 12%, and that these the values are significant (*p* < 0.05) (Table 1). In addition, for Q in the same range of concentration the intensity of MC540 fluorescence was much more reduced, i.e., within the range 29–56%, and the values are significant (*p* < 0.05) too. The fluorescence intensity of MC540 is also proportional to the molecular packing of lipids in the bilayer. The lower the fluorescence, the greater the packing order [30].

On the basis of the changes in fluorescence anisotropy of the probes DPH and TMA-DPH, the impact of Q 7-MeGlu and Q on the fluidity of the hydrophobic regions of the lipid membrane was determined. The TMA-DPH probe, located at the level of the fourth carbon atom in the alkyl chain, provided information on the exact location of the modifier molecule between the hydrophobic and hydrophilic parts of the membrane bilayer [31], while the DPH molecules can be placed at various locations in the non-polar tail region of the membrane, and thus be useful to study the entire organization and dynamics of cell membranes and liposome membranes. Its anisotropy correlates with the probe’s rotational diffusion movement, and by being distributed throughout the bilayer, it proved to be extremely sensitive to the packing order of the lipid chains and the membrane fluidity [32]. The results showed that Q 7-MeGlu and Q are responsible, in a concentration-dependent way, for increasing the anisotropy for both the probes. Q 7-MeGlu caused an increase in hydrophobic anisotropy in the probe DPH from ca. 6 to 51% in comparison to control; furthermore, Q was responsible for increasing the anisotropy of the probe from 29 to 97% under identical concentrations (*p* < 0.05). Relative changes in anisotropy in the probe TMA-DPH were caused by the interaction between Q 7-MeGlu, as well as Q, with the membrane, were observed within the range 5–10% and 16–37%, respectively, the values being significant (*p* < 0.05). The observed increase in anisotropy in the probes DPH and TMA-DPH proved that the probes’ micro-surrounding became more rigid, i.e., a decrease in its fluidity was observed [33]. In scientific reports there is also a similar opinion that in the case of quercetin its impact on lipid membranes can indicate limited movements of hydrocarbon chains in the hydrophobic regions as well as increase in ordering of the lipid heads in their hydrophilic regions of the lipid bilayer [34,35,36].

Compared to quercetin, the Q 7-MeGlu molecule without a hydroxyl group at the 7th position showed less membrane interactivity, supporting the importance of the 7-hydroxyl group in the A ring. A similar trend was noticed by Tsuchiya [31], who compared quercetin to quercetin-3-rutinoside and quercetin-3-glucoside. He proved that the membrane interactivity disappeared when the hydroxyl group was replaced by the glucoside group. There was much more limited interaction between Q with linked glycoside moiety and lipid membrane since the sugar moiety abolished the hydrophobicity and also rendered this molecule larger in size.

The location of natural polyphenols, e.g., flavonoids, in membranes remains a matter of controversy. Some experimental studies suggest that flavonoids with numerous polar OH groups (e.g., quercetin and catechin) are located deeply inside membranes. Other studies locate flavonoids closer to the membrane surface where the interaction with the polar head-groups of lipids via H-bonding is observed. The location and orientation of flavonoids affects membrane structure and function, in particular fluidity, ability to conduct ions and volume. The location of flavonoids inside membranes depends strongly on pH and flavonoid charge; the lower the pH, the lower the deprotonation state and the deeper the penetration [37]. The presented results, which were obtained using several fluorometric probes localized at different depth of lipid bilayer showed that Q 7-MeGlu is able to exert influence not only on membrane surfaces by interaction with phospholipid polar heads, but also on deeper regions—on the interface and upper hydrophobic regions of phospholipid acyl chains.

#### 2.2.2. ^1^H-NMR Study

**Figure 1:**
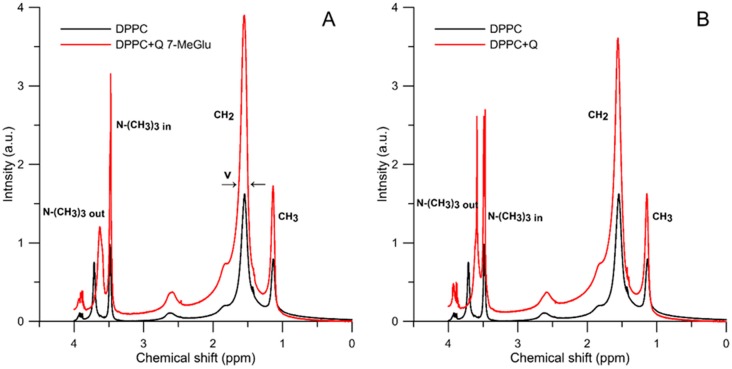
^1^H-NMR spectra of liposomes (at 325 K) formed from pure DPPC (36 mM) and DPPC with (**A**) quercetin 7-*O*-β-d-(4″-*O*-methyl)glucopyranoside (Q 7-MeGlu) and (**B**) quercetin (Q) at concentration 0.24 mM. PrCl_3_ (4 mM) was added to the samples before measurement.

The solid state proton NMR technique was also employed to monitor the effects of the interaction between the tested flavones and membranes. The applicability of this technique in membrane structure studies is associated with the fact that the shape of NMR signals depends on the motional dynamics of the molecular fragments (comprising particular nuclei being in resonance) [38]. Figure 1 presents the ^1^H-NMR spectra of DPPC liposomes after addition (at 36 mM concentration) of Q 7-MeGlu (Figure 1A) and Q (Figure 1B). The concentration of Q 7-MeGlu and Q was 0.24 mM (i.e., the ratio DPPC to Q 7-MeGlu or Q was 150:1, *v*/*v*).

Several bands are visible in the spectra that correspond to the following major molecular features of DPPC membranes: -CH_3_ and -CH_2_ groups of the hydrophobic region of the membrane as well as the bands of -N^+^-(CH_3_)_3_ from the membrane polar head region of the membrane. Adding praseodymium cations (Pr^3+^) results in a split of the ^1^H-NMR band corresponding to the ammonium group (Δδ) into two signals, one coming from the outer leaflets of the liposome membranes (-N^+^-(CH_3_)_3_)_out_) and second from the inner liposome surface (-N^+^-(CH_3_)_3_)_in_).

The ratio of the areas under the signal assigned to the outer layer I_Out_ to the inner layer I_In_ is in proportion to the number of DPPC molecules in the outer and inner layers. For small unilamellar liposomes the ratio I_out_/I_in_ is greater than 1 [39,40].

Adding Q 7-MeGlu or Q leads to a series of changes in the spectra; parameters (Table 2): in full width at half height (ν) of the ^1^H-NMR bands, in the ratio I_out_/I_in_ and in the splitting (Δδ) of the band corresponding to the ammonium group -N^+^-(CH_3_)_3_. A significant increase in the full width at half height (ν) of the ^1^H-NMR bands after adding Q 7-MeGlu or Q was observed (Figure 1A,B). In this case, an increase in the half width was observed to be approximately 119% and 142% in the presence of Q and Q 7-MeGlu, respectively. Such a large broadening of the membrane surface signal is associated with a high reduction in mobility of these groups due to the incorporation of the Q molecules or its derivatives into the membrane. In other words, a relatively high ordering effect in the hydrophilic region (restriction of motional freedom) of the membrane in the presence both sugar derivatives of quercetin as well as aglycone were observed. The greater signal broadening due to incorporation into the membrane of Q 7-MeGlu than Q is probably the result of the more hydrophilic nature of these molecules. Moreover, molecular dynamic simulations demonstrate that flavonoids (quercetin and its metabolite) are concentrated at the lipid bilayer/water interface, just below the membrane surface. Insertion of non-polar groups increases the penetration depth (methoxy derivatives) while insertion of polar groups decreases the penetration depth (sulphate, glucuronide etc.) [34]. However, Q particles can locate deeper in the polar region of the membrane [36,41,42]. Both tested flavones after incorporating into the lipid membrane are able to modify the lipid membrane properties also due to the fact that hydrogen bonds are likely to be formed. Quercetin was proved to bind to the membrane surface via hydrogen bonds at the lipid/water interface [37,43,44]. The small increase (approx. 5%) in the case of –CH_2_ group was observed after incorporating both compounds into membrane. At the same time, a slight decrease (4%) in full width at half height (ν) of band of terminal methyl group in ^1^H-NMR spectrum in the presence of Q was observed. Moreover, the presence of Q clearly increased the I_out_/I_in_ ratio, from 0.9977 in pure DPPC to 1.3305 in liposomes (33%) while addition of Q 7-MeGlu slightly decreased this parameter by 13% (Table 2). This means that the effect of small unilamellar liposome formation in the presence of Q was observed (I_out_/I_in_ = 1.3305) and not entirely homogeneous liposome formation in the presence of Q 7-MeGlu (I_ou_t/I_in_ = 0.8646). Q or Q 7-MeGlu addition also resulted in 32% and 56% decrease (Δδ) in the splitting parameter of the resonance maximum corresponding to polar head groups, respectively. This probably implies that the relatively strong effect of membrane surface ordering and the altered distribution of local charges on the membrane surface by intercalation of flavones molecules limit the penetration of Pr^3+^ ions into the membrane (Q 7-MeGlu > Q).

The effect of Q 7-MeGlu/Q on the hydrophilic region of DPPC liposomes (compared with PC) connected with increased order of that region is close to results obtained with MC540 probe. Nonetheless, the small reduction in mobility of hydrocarbon chains due to the effect of Q 7-MeGlu/Q on the hydrophobic region of DPPC liposome membrane may relate to the effect which was induced by Q 7-MeGlu/Q on the vicinity of DPH probe. We think that the Q 7-MeGlu/Q incorporate into PC and DPPC membranes may follow from similarity of their affinity. The tighter packing of lipids in DPPC membrane (compared with PC) may result in “shallower” incorporation of a certain batch of molecules into the hydrophilic region of membrane, causing local structural deformations that enable other molecules to intercalate the membrane deeper. As a result, one can expect a certain perpendicular distribution of incorporated molecules in a DPPC membrane. Studies by Scheidt et al. [45] e.g., using modern ^1^H MAS NMR techniques on location of selected flavonoids, including luteolin-7-glucoside in POPC membranes (monousaturated zwiterionic lipid) showed that those flavonoids are still distributed over the entire membrane to protect any given double bonds from oxidation. 

^1^H-NMR method the knowledge of the large limitations (stiffening effect) in the segmental movement (ammonium moiety segment) by both the flavones at the outer monolayer lipid molecules (Q 7-MeGlu > Q) and most likely a small mobility restriction in the hydrophobic core of the lipid membrane are extensive.

### 2.3. Liposome Oxidation Assay

**Figure 2:**
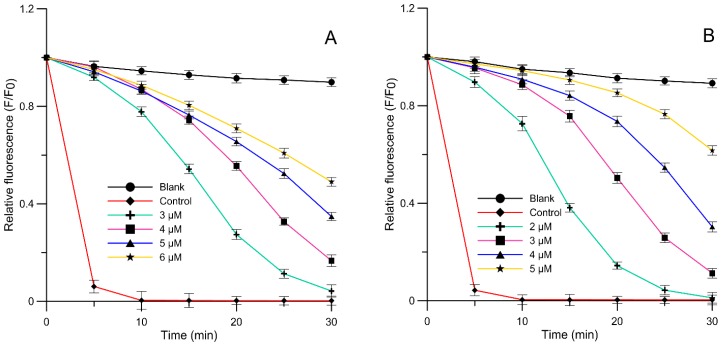
Relative fluorescence intensity of DPH-PA probe as a function of oxidation time for PC membranes and AAPH radicals in the presence of (**A**) quercetin 7-*O*-β-d-(4″-*O*-methyl) glucopyranoside and (**B**) quercetin, for several concentrations. The relative changes in fluorescence intensity *F*/*F*_0_ are a measure of the degree of lipid peroxidation (*F*_0_—fluorescence in the control sample (oxidized by AAPH without study compounds), *F*—fluorescence of samples in the presence of study molecules). Blank sample -not oxidized by AAPH and without study compounds.

The antioxidative activity of Q 7-MeGlu and Q was tested using the fluorometric method on the assumption that that ability was due to by free radicals induced in thermal decomposition in AAPH compound within phosphatidylcholine liposome membrane. Free radicals induced by AAPH oxidized the probe DPH-PA decreasing its fluorescence. As a measure of lipid-membrane oxidation was assumed relative fluorescence intensity, calculated as the ratio of probe’s fluorescence intensity to initial/control value of the intensity. The studied compounds by scavenging free radicals decreased DPH-PA fluorescence intensity. Some examples of the kinetic curves of relative fluorescence intensity for the probe DPH-PA in the presence of Q 7-MeGlu or Q, in phosphatidylcholine liposome membrane are shown in Figure 2A,B, respectively. The presented figures prove that the fluorescence intensity increases in a concentration-dependent way for Q 7-MeGlu or Q, marking an increase which is in proportion towards this concentration in line with inhibition of the Q 7-MeGlu or Q oxidative process in the lipid membranes. The plot of the relation between the degree of oxidation inhibition versus Q 7-MeGlu or Q concentrations was the basis to specify the IC_50_ parameter (linear equations *R*^2^ = 0.9867 and *R*^2^ = 0.9903, respectively). The values of this parameter are presented in Table 3; e.g., in comparison with L-(+)-ascorbic acid. Both compounds are effective in lipid membrane protection against free radicals induced by AAPH. Only a slight difference in IC_50_ of Q and Q 7-MeGlu is observed (Q ≥ Q 7-MeGlu), the values are significant (*p* < 0.05). In particular, Q 7-MeGlu showed ca. 1.2-fold lower activity than quercetin and ca. 24-fold higher antioxidative activity than L-(+)-ascorbic acid (Table 3).

The high antioxidative activity of both flavanols—Q 7-MeGlu and Q—is caused by their molecular structure. The core of it is the presence of the *ortho*-dihydroxylation of the B ring, the 2,3-double bond in conjugation with a 4-oxo function, and the presence of both 3- and 5-hydroxyl groups [48]. Slightly lower activity of Q 7-MeGlu in comparison to Q in the protection of membrane lipids against the process of peroxidation may be caused by blocking the hydroxyl group bound to carbon C7 in ring A. The majority of reports referring to the importance of substituents in the molecular structures of flavonoids prove that in vitro aglycones show higher antioxidative activity than their glycosylated forms. Moreover, an increase in the number of sugar substituents leads to a decrease in antioxidative activity [7,49]. A limited number of reports focused on the interaction between flavonoids and model membranes prove that there is a link between intercalation of flavonoids to the membranes and their effectiveness in protection against free radicals [12,33,44,45]. The results of the research presented in this paper which were collected using fluorometric probes and ^1^H-NMR methods allow to indicate that there is a connection between the ability of Q 7-MeGlu and quercetin to protect phospholipid liposomes membranes against peroxidation and their location and structural changes induced in the membrane. In our opinion, the partial decrease in lipid peroxidation inhibition observed in case of Q 7-MeGlu compared to quercetin can be explained by their molecular location and orientation in the lipid bilayer. A more hydrophilic substituent of Q 7-MeGlu (in comparison with quercetin) indicates that its location is mainly in the polar region of membrane and that explains its weaker inhibition activity. It can be also assumed that Q 7-MeGlu (and also Q) show antioxidative activity not only due to their radical scavenging properties, but also by rigidifying the membrane. Such a location and ordering of lipids are likely to reduce the concentration of free radicals within the membrane and can be a kind of barrier against those free radicals which penetrate into the membrane surface. By ordering and sealing the membrane with molecules Q 7-MeGlu or Q bound to the lipid polar heads, it is possible to change their hydration and consequently to inhibit the infusion of free radicals into the membrane.

### 2.4. Inhibition of 1 and 2 Cyclooxygenases

The potential anti-inflammatory activity of Q 7-MeGlu and e.g., Q was proved on the basis of inhibition of the cyclooxygenase (COX-1, COX-2) activity. Cyclooxygenase 1 and 2 participate in the synthesis of arachidonic acid, prostaglandin and leukotrienes—the mediators of inflammation [50]. As a result of those enzymes’ inhibition caused by flavonoids, the synthesis of prostaglandin PGE2, leukotriene B_4_ and thromboxane A_2,_ which are responsible for inhibiting leukocyte accumulation and adjusting the vascular system, is decreased, and therefore the overall inflammation is reduced. Our studies proved that Q 7-MeGlu is able to inhibit by about 50% the activity of COX-1 and COX-2 a concentration of 45.06 and 49.23 µM, respectively. Values of parameter IC_50_ for anti-inflammatory medicine are IC_50_ = 25.57 and IC_50_ = 21.25 µM for COX-1 and COX-2, respectively, (Table 3). The data referring to the anti-inflammatory activity of Q 7-MeGlu in the process of COX-1 and COX-2 inhibition still have not been published. However, the anti-inflammatory activity of quercetin, which is the initial substrate of the biotransformation, has already been proved. Q anti-inflammatory activity is based on blocking activation of kinases p38, MAPK, Akt and transcription factor NF-κB [51]. Our comparative studies using the non-steroidal anti-inflammatory drug (NSAIDs): indomethacin (Table 3) showed that it is responsible for inhibiting pro-inflammatory enzymes at a concentration significantly lower than Q 7-MeGlu. However, despite their significant therapeutic efficiency, NSAIDs can cause side effects, e.g., gastrointestinal bleeding, cardiovascular side and suppressed function of the immune system. That is why the attention is focused on natural pharmacotherapies. Our research showed that Q 7-MeGlu as a COX inhibitor can potentially be developed into a much more safer and effective compound for the treatment of inflammation, since it can potentially inhibit biosynthesis of both prostaglandins and leukotrienes from arachidonic acid and eliminate the harmful effects of NSAIDs.

### 2.5. Binding to Human Serum Albumin (HSA)

Human serum albumin is the most abundant plasma protein in the circulatory system and is responsible for the binding and transporting of many endogenous and exogenous substances including hormones, fatty acids and drugs. The interaction of flavonoids with serum albumin may significantly affect the most important biological properties of those compounds, *inter alia*: distribution, metabolism, excretion and toxicity in vivo [52].

**Figure 3:**
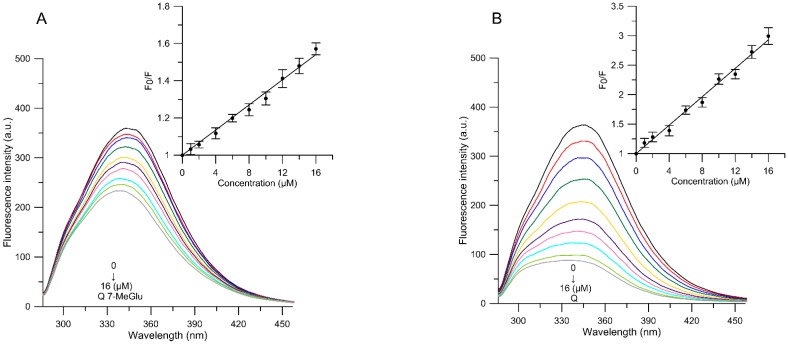
Emission spectra of HSA in the presence of various concentrations of (**A**) quercetin 7-*O*-β-d-(4″-*O*-methyl)glucopyranoside (Q 7-MeGlu); (**B**) quercetin (Q), and Stern-Volmer plots of *F*_0_/*F* against concentration of studied molecules (HSA = 1.5 × 10^-5^ M, *λ*_ex_ = 280 nm, *T* = 310 K).

In order to demonstrate the Q 7-MeGlu and Q binding to HSA, the HSA fluorescence emission spectra in the absence or presence of studied compounds were analyzed. The fluorescence emission spectra of the compounds–HSA system were measured at four different temperatures (300, 305, 310, 315 K). The effect of tested flavonoids on fluorescence intensity of HSA at 305 K is shown in Figure 3A,B. HSA had a strong fluorescence emission at ca. 345 nm after it had been excited at a wavelength of 280 nm. When Q 7-MeGlu and Q of 1-16 µM concentration were added to the HSA solution, the HSA fluorescence intensity decreased regularly in accordance with the studied compounds’ concentration. This result suggests that analyzed molecules are able to interact with HSA and quench its intrinsic fluorescence.

Fluorescence quenching induced by studied molecules is described using the well-known Stern-Volmer equation [53]:

(1) $$\frac{F_{0}}{F}=1+K_{q}\tau_{0}\left[Q_{u}\right]=1+K_{SV}\left[Q_{u}\right]$$

where: *F*_0_ and *F* are HSA fluorescence intensities before and after adding quencher, respectively, *K_q_* is a bimolecular quenching constant, *𝜏_0_* is life time of the fluorophore in the absence of quencher (the fluorescence life time of a biopolymer is about 5 × 10^−9^ s [54], [Q_u_] is the quencher concentration, and *K_SV_* is the Stern–Volmer quenching constant (*K_SV_ = Kq ×* *𝜏*_0_). The plot of *F*_0_/*F* against [Q_u_] at 310 K is shown in Figure 3A,B. The Stern–Volmer plots are evidently linear in the 1–16 μM range of concentration for studied compounds.

Furthermore, in order to explain (and to compare with quercetin) the mechanism of the Q 7-MeGlu binding to HSA, the temperature dependence of the quenching constants (*K*_SV_) were studied. As shown in Table 4, the values of *K_SV_* decreased when the temperature increased. The study showed that the presence of 4-*O*-methyl group in glucopyranose moiety in the A ring of quercetin influenced the binding to HSA. *K_SV_* for Q 7-MeGlu was approximately four times lower than *K_SV_* for Q. The same tendency was also observed for different flavonoids, where the glycosyl and 4-*O*-methyl group in glucopyranose moiety reduced the affinity of flavonoids for human serum albumin [19,28].

Fluorescence quenching can be accounted for by two interaction mechanisms, also known as dynamic or static quenching. According to reports, dynamic quenching, which is caused by collisions and higher temperatures, results in larger diffusion coefficients. In static quenching, increased temperature is likely to destabilize the bound complexes, and thus, lower values of the static quenching constants are expected [52]. Our results suggest that the mechanism of HSA quenching by studied molecules was likely to be initiated by complex formation. Moreover, the values of the quenching rate constant *K_q_* (calculated using the equality $K_{q}=K_{sv}/\tau_{0}$) were all far greater than the limiting diffusion constant of the biomacromolecule (1.0 × 10^10^ M^−1^ s^−1^). The values of *K_q_* for Q 7-MeGlu binding to HSA, e.g., at 310 K, was calculated to be 6.824 × 10^12^ and 31.264 × 10^12^ M^−1^·s^−1^, respectively. Taking into consideration the above experimental results and analysis, it may be concluded that the HSA fluorescence quenching may originate from the formation of Q 7-MeGlu–HSA and Q–HSA complex via a static quenching process.

The value of the binding constant *K_a_* is crucial for understanding the distribution of the drug in plasma. A strong binding produces a decrease in plasma concentrations, improving the distribution and the pharmacological effect of the compound [55]. The apparent binding constant (*Kb*) and number of binding sites (*n*) can be calculated using the following equation:

(2) $$log\left(\frac{F_{0}-F}{F}\right)=logK_{b}+nlog\left[Q_{u}\right]$$

The values of *n* and *K_b_* were obtained by plotting log [(*F*_0_ *− F)/F*] versus log[*Q_u_*]. The results for Q 7-MeGlu and aglycone as Q_u_ (as the quencher) at four different temperatures (300, 305, 310, and 315 K) are presented in Table 4. The results showed that the values of *K_b_* decreased when the temperature increased, implying that the complex of studied molecules with HSA became unstable when the temperature increased. Binding parameters to human serum albumin for quercetin are in accordance with scientific reports [52,56]. This is demonstrated by the fact that the Q 7-MeGlu complex shows weaker binding to HSA than to Q-HSA complex (Table 4). The lower binding constant of the complex to Q 7-MeGlu-HSA can be explained by structure-binding affinity relationships that showed that glycosylation of the flavonoid produced steric hindrance in the binding pocket, which weakened the binding affinity and increased the polarity of the molecule, thereby lessening the flavonoid ability to penetrate into the tryptophan-rich hydrophobic interior regions of HSA [57]. In addition, numerous studies proved that drugs usually bind to high-affinity sites with typical association constants in the range of 10^4^–10^6^ M^−1^. In particular, Wilgusz et al. [58] proved that the anti-inflammatory drug of third generation meloxicam is able to bind to HSA in more than 99% with association constant of the order of 10^5^ M^−1^; furthermore, aspirin and indomethacin binding constant to HSA–aspirin/indometacin is approximately 1.4 × 10^4^ and 48.9 × 10^4^ M^−1^, respectively [59]. The range of binding constants for Q 7-MeGlu and Q to HSA, as obtained in the present study, indicates a relatively good interaction between sugar derivatives of quercetin and its aglycon and HSA. The calculated number of binding sites (*n* = 1.082–0.437 for Q 7-MeGlu and *n* = 1.048–0.645 for Q), of approximately 1.0 (except for the possible 1:2 complex for Q 7-MeGlu-HSA at *T* = 315 K), indicate that there is only one binding site in the HSA molecule at different temperatures.

The interaction forces between a drug and a biomacromolecule may include hydrophobic forces, electrostatic interactions, van der Waals interactions, hydrogen bonds, etc. The thermodynamic parameters of binding reaction are the main evidence for confirming a binding force. In order to elucidate the binding mode, the binding constant for flavonoid–HSA complex formation was evaluated at four different temperatures. The thermodynamic parameters of the binding process can be calculated using the Van’t Hoff equation [60]:

(3) $$lnK_{b}=\frac{-\Delta H}{RT}+\frac{\Delta S}{R}$$

The enthalpy change ∆*H* is calculated on the basis of the slope of *lnK_b_* vs. Δ*H* plot. The free-energy change Δ*G* is estimated on the basis of the following equation:

(4) $$\Delta G=\Delta H-T\Delta S=-RTlnK_{b}$$

where: Δ*H* Δ*G* and Δ*S* are enthalpy, free enthalpy and entropy change, respectively. *R* is the gas constant 8.314 J·mol^−1^·K^−1^.

The negative values of ΔG support the spontaneous nature of the drug binding process to the proteins. When Δ*H* < 0 and Δ*S* > *0*, the electrostatic force dominates the interaction; when Δ*H* < 0 and Δ*S* < 0, van der Waals interactions and hydrogen bonds dominate the reaction and when Δ*H* > 0 and Δ*S* > 0, hydrophobic interactions dominate the binding process [61].

The values of ∆*G*, ∆*H* and ∆*S* in our study, which are obtained for Q 7-MeGlu and Q at different temperatures, are shown in Table 4. It may be noted that the Δ*G* values were negative for Q 7-MeGlu and Q, which indicates that the interaction process was spontaneous. The negative ∆*H* and ∆*S* values for quercetin derivatives and its aglycone suggest that the compounds bind to HSA mainly as a result of hydrogen bonds and van der Waals forces.

## 3. Materials and Methods

### 3.1. General Information

Reagents and solvents (analytical or HPLC grade) were purchased from Sigma-Aldrich (St. Louis, MO, USA), Merck (Darmstadt, Germany) or POCH (Gliwice, Poland). TLC was carried out on Merck silica gel 60 F254 (0.2 mm thick) plates with solvent mixture acetone:chloroform:hexane:formic acid in H_2_O (15% *v*/*v*) (35:25:2:1, *v*/*v*). Spots were visualized under short- and long-wavelength UV light, plates were then sprayed with methanol–sulfuric acid (1:1, *v*/*v*) solution. HPLC was performed on an Ultimate 3000 UHPLC^+^ focused instrument (Thermo Scientific, Waltham, MA, USA) with a photodiode array detector (detection from 210 to 450 nm wavelength) using a ZORBAX Eclipse XDB analytical HPLC column (5 µm, 4.6 mm × 250 mm, Agilent, Santa Clara, CA, USA) at the flow rate of 1.2 mL/min and the following elution program: isocratic elution from 0 to 2 min (80% A); gradient elution: from 2 to 8 min (80% A → 70% A), from 8 to 10 min (70% A → 60% A), from 10 to 16 min (60% A → 5% A), isocratic elution: from 16 to 20 min (5% A), gradient elution: from 20 to 21 min (5% A → 80% A). As a solvent A 0.05% HCOOH in water and as a solvent B 0.05% HCOOH in MeCN were used. The biotransformation product was purified by means of a PuriFlash PF430 flash chromatography system (Interchim, Montluçon, France), using an Interchim PF-30SIHP-F0025-30 µm flash column with isocratic elution of an acetone:chloroform:hexane:formic acid in H_2_O (15% *v*/*v*) (35:25:2:1, *v*/*v*) solvent mixture at the flow rate 15 mL/min and detection at *λ* = 255 nm and *λ* = 370 nm wavelength. UV spectra were recorded on a Cintra 303 spectrophotometer (GBC Scientific Equipment, Braeside, Australia) in methanol. ^1^H-NMR, ^13^C-NMR, DEPT 135°, ^1^H–^1^H NMR (COSY), and ^1^H–^13^C NMR (HSQC) spectra were recorded on a DRX Avance II 600 (600 MHz) instrument (Bruker, Billerica, MA, USA) in DMSO-*d*_6_. Negative-ion HR ESI-MS spectra were registered on a Bruker microTOF-Q spectrometer using direct infusion with the following parameters: the mass spectrometer was operated in negative ion mode with the potential between the spray needle and the orifice 4.5 kV, nebulizer pressure of 0.4 bar, and a drying gas flow rate of 4 L/min at 200 °C. The sample flow rate was 180 µL/min. Ionization mass spectra were collected at the ranges *m*/*z* 150–3000. The instrument was calibrated with an Agilent electrospray calibration solution (ESI-L low concentration Tuning Mix-Agilent Technologies, Agilent Product Number: G1969-85000) that was diluted with acetonitrile.

### 3.2. Materials

2,2′-Azobis(2-amidinopropane) dihydrochloride (AAPH), human serum albumin (HSA) (lyophilized powder, essentially fatty acid free), indomethacin, *N*,*N*,*N’*,*N’*-tetramethyl-*p*-phenylenediamine (TMPD), arachidonic acid from porcine liver, cyclooxygenase 1 from sheep, cyclooxygenase 2 human recombinant, 1,2-dipalmitoyl-sn-glycero-3-phosphatidylcholine (DPPC), l-α-Phosphatidylcholine (from egg yolk) were purchased from Sigma–Aldrich. The probes 1,6-diphenyl-1,3,5-hexatriene (DPH), 3-[*p*-(6-phenyl)-1,3,5-hexatrienyl]propionic acid (DPH-PA), Merocyanine 540 (MC540), and *N*,*N*,*N*-trimethyl-4-(6-phenyl-1,3,5-hexatrien-1-yl)phenylammonium *p*-toluenesulfonate (TMA-DPH) were purchased from Molecular Probes (Eugene, OR, USA). Tris (hydroxymethyl) aminomethane (Tris:HCl) were obtained from “Chempur” (Piekary Śląskie, Poland). Quercetin (Q) was purchased from Sigma-Aldrich. Yellow powder, ^1^H-NMR (DMSO-*d*_6_) δ_H_: 6.19 (1H, d, *J* = 2.1 Hz, H-6), 6.41 (1H, d, *J* = 2.1 Hz, H-8), 6.89 (1H, d, *J* = 8.5 Hz, H-5′), 7.54 (1H, dd, *J* = 8.5, 2.2 Hz, H-6′), 7.68 (1H, d, *J* = 2.2 Hz, H-2′). ^13^C-NMR (DMSO-*d*_6_) δ_C_: 93.2 (C-8), 98.0 (C-6), 102.9 (C-10), 114.9 (C-2′), 115.4 (C-5′), 119.8 (C-6′), 121.8 (C-1′), 135.6 (C-3), 144.9 (C-3′), 146.6 (C-2), 147.5 (C-4′), 156.0 (C-9), 160.6 (C-5), 163.7 (C-7), 175.7 (C=O).

### 3.3. Microorganism

The strain *B. bassiana* AM278 used for the biotransformation was purchased from the Institute of Biology and Botany of Wrocław Medical University (Wrocław, Poland). It was maintained on agar slants at 5 °C and grown on a Sabouraud medium (3% glucose and 1% peptone) at 25 °C.

### 3.4. Conditions for Biotransformations

The culture was shaken on rotatory shaker (130 speed, 7 amplitude) at 28 °C in 300 mL Erlenmeyer flasks with 100 mL of the Sabouraud medium. Agar slant culture was used to obtain the pre-culture (100 mL Erlenmeyer flasks with 30 mL of the medium) and then 3-day pre-culture was transferred to the main culture media. Substrate was added after 7 days of culturing. 240 mg of substrate Q dissolved in 8 mL of DMSO was equally distributed among 8 flasks (1 mL each). The reactions were carried until the substrate was metabolized (the progress of conversion was monitored by HPLC). Then the cultures were acidified with 1 M HCl to pH around 5 (if necessary) and reaction mixtures were extracted with ethyl acetate (three times × 50 mL). Obtained extract was dried over anhydrous magnesium sulphate, filtered and evaporated under vacuum. Substrate stability control consisted of the quercetin (Q) dissolved in DMSO and a sterile growth medium incubated without microorganisms.

### 3.5. Products Isolation

The biotransformation products were separated using the Interchim PuriFlash PF430 Flash Chromatography System. Product structure was elucidated by NMR and MS spectroscopy methods. Quercetin 7-*O*-β-d-(4″-*O*-methyl)glucopyranoside (Q 7-MeGlu) was obtained by biotransformation of quercetin (Q) catalyzed by *Beauveria bassiana* AM278 after 11 day biotransformation with yield 15.6% (53 mg). Yellow powder, ^1^H-NMR (DMSO-*d*_6_) δ_H_: 3.05 (1H, t, *J* = 9.0 Hz, H-4″), 3.26 (1H, dd, *J* = 9.0, 7.8 Hz, H-2″), 3.44 (1H, t, *J* = 9.1 Hz, H-3″), 3.46 (3H, s, C4″-OCH_3_), 3.49–3.53 (1H, m, C-5″ overlapped on H-6″a), 3.49–3.53 (1H, m, H-1″a, overlapped on H-5″), 3.62–3.68 (1H, m, H-6″b), 5.10 (1H, d, *J* = 7.8 Hz, H-1″), 6.42 (1H, d, *J* = 2.2 Hz, H-6), 6.76 (1H, d, *J* = 2.2 Hz, H-8), 6.90 (1H, d, *J* = 8.5 Hz, H-5’), 7.55 (1H, dd, *J* = 8.4, 2.2 Hz, H-6′), 7.72 (1H, d, *J* = 2.2 Hz, H-5’).^13^C NMR (DMSO-*d*_6_) δ_C_: 59.7 (C-4″-OCH_3_), 60.1 (C-6″), 73.2 (C-2″), 75.6 (C-5″), 76.0 (C-3″), 78.8 (C-4″), 94.2 (C-8), 98.6 (C-6), 99.5 (C-1″), 115.3 (C-2’), 115.5 (C-5’), 120.0 (C-6’), 121.8 (C-1’), 136.0 (C-3), 145.0 (C-3’), 147.50 (C-2), 147.8 (C-4’), 155.7 (C-9), 160.1 (C-5) 162.6 (C-7), 175.9 (C=O).

### 3.6. Biological Activity Study Methods 

#### 3.6.1. Liposome Preparation

Small unilamellar liposomes (SUV) consisted of phosphatidylcholine (egg-PC) were prepared in accordance with the methodology described by Gabrielska and Oszmiański [62]. The lipids were dissolved in chloroform (100 mg/mL), evaporated to dryness under nitrogen and under vacuum for another 60 min. Subsequently, a phosphate buffer (Tris-HCl) of pH 7.4 was added and liposomes were formed by mechanical shaking. Then SUVs were formed using a 20 kHz sonicator for 15 min. During sonication the samples were thermostated at 0–2 °C. In the study, PC liposomes at a concentration of 0.1 mg/mL in phosphate buffer (pH 7.4) were used. Liposomes prepared according to the presented-above procedure were used to determine the antioxidative activity and in fluorometric studies.

#### 3.6.2. Fluorescence Measurements—MC540, TMA-DPH and DPH Probes

The Q 7-MeGlu and Q effect on the hydrophilic and hydrophobic properties of the membrane was determined using the fluorometric method applying the MC540, TMA-DPH and DPH fluorescence probes according to the procedure described by Strugała et al. [46,47]. Merocyanine 540 (MC540) is a probe that partitions into the membrane and orients parallel to the glycerol backbones of the lipids [63]. The fluorescence intensity of MC540 emission was determined and expressed as relative intensity change in relation to control as a function of studied compounds concentration. The TMA-DPH and DPH probes are supposed to distribute in the hydrophobic part of the bilayer. All the fluorescent probes were used at 1 μM concentration. The DPH and TMA-DPH stock solutions were prepared in DMF, while the probe MC540 was dissolved in methanol. Liposomes were prepared according to procedure described in Section 3.6.1. The control samples contained liposomes and a fluorescent probe, while the study samples contained compounds (Q 7-MeGlu or Q at final concentrations 1, 5 and 10 µM). Liposomes suspended in buffer were incubated for 30 min with probe. In next step, Q 7-MeGlu or Q were added at final concentrations 1, 5 and 10 μM and incubated for 10 min. The excitation and emission wavelengths were as follows: for MC540 λ_ex_ = 540 nm and λ_em_ = 590 nm, for TMA-DPH λ_ex_ = 340 nm and λ_em_ = 430 nm, and for DPH probe λ_ex_ = 360 nm and λ_em_ = 425 nm. Fluorescence anisotropy for TMA-DPH and DPH was calculated using the following equation [64]:

(5) $$A=\frac{I_{∥}-GI_{⊥}}{I_{∥}+2GI_{⊥}}$$

where $I_{∥}$ and $I_{⊥}$ are fluorescence intensities observed in directions parallel and perpendicular, respectively to the polarization plane of the exciting wave. G is the apparatus constant dependent on the emission wavelength.

The fluorescence experiment was conducted using a Cary Eclipse fluorimeter (Varian, Palo Alto, CA, USA). Measurements were carried out at room temperature (approx. 20 °C). The experiment was performed in six replicates (*n* = 6).

#### 3.6.3. ^1^H-NMR Studies

The ^1^H-NMR method of investigating the Q 7-MeGlu and Q interaction with liposomes was described earlier by Gabrielska et al. [65] with minor modifications [47]. Mixtures of phospholipids (DPPC) with studied compounds were co-dissolved in a chloroform/methanol mixture (55:1 *v*/*v*) at the respective concentrations. The lipid concentration in the sample was 36 mM and that of Q 7-MeGlu and Q was 0.24 mM (150:1 *v*/*v*). The samples were first evaporated under a stream of nitrogen and then in a vacuum (overnight). Then the samples were hydrated with D_2_O and vigorously shaken (15 min) at temperature above the main phase transition of lipid (41 °C) until optical homogeneity of the mixture was reached. Next, the lipid suspension was sonicated with a 20 kHz sonicator (20 kHz, Sonic, Italy) to yield a homogeneous lipid dispersion. Briefly, before the measurements a 4 mM praseodymium trichloride (PrCl_3_ × 6H_2_O) in heavy water solution (6 µM) was added to the sample of 0.6 mL liposome suspension. ^1^H-NMR parameters were as follows: spectral window 12.019 Hz, digital resolution 0.183 Hz, acquisition and delay times 2.73 s and 1.00 s, respectively, and acquisition temperature 325 K. The experiment was performed in two replicates.

#### 3.6.4. Liposome Oxidation Assay

Antioxidant activities of Q 7-MeGlu and Q were determined using a fluorometric method described earlier by Strugała et al. [47] with minor modifications. The studies were carried out on PC liposomes, which contained the fluorescent probe DPH-PA. Use was made of the relationship between DPH-PA fluorescence intensity and concentration of free radicals. The probe’s fluorescence decreased with its rising oxidation caused by free radicals, supplied by AAPH. Molecules of this compound underwent thermal decomposition into two free radicals each. The value of relative intensity of DPH-PA fluorescence was adopted as a measure of the degree of lipid membrane oxidation [66]. It was calculated as a ratio of fluorescence intensity after 30 min of oxidation in the presence of antioxidants to the initial value of the intensity. The studied polyphenolic compounds scavenged free radicals and thus caused a lower rate of DPH-PA fluorescence decrease. PC liposomes at a concentration of 0.1 mg/mL in phosphate buffer (pH 7.4) were used, incubated for 0.5 h in darkness with addition of DPH-PA probe at a concentration of 1 µM (stock solutions were prepared in DMF). Concentration of DMF in a sample did not exceed 0.16%. The wavelengths of excitation and emission for the probe were as follows: λ_ex_ = 355 nm, λ_em_ = 430 nm. Oxidation was initiated just before measurement, using AAPH at a concentration of 1 M (dissolved in water) at 37 °C (control sample), or in the presence of test substances (Q 7-MeGlu and Q). The concentrations of studied compounds fluctuated in the range 3–7 µM for Q 7-MeGlu and 2–5 µM for Q. The measurements were conducted with a Cary Eclipse fluorimeter. The percentage inhibition of lipid oxidation was calculated on the basis of the following formula:

(6) $$\%\;inhibition=\;\frac{\left(F_{S}-F_{C}\right)}{\left(F_{B}-F_{C}\right)}\cdot 100\%$$

where: *F_S_* is relative fluorescence of probe oxidized with AAPH in the presence of antioxidant, *F_C_* is relative fluorescence of control sample oxidized with AAPH without antioxidant, *F_B_* is relative fluorescence of the blank sample (not oxidized by AAPH and without antioxidant).

#### 3.6.5. Inhibition of Cyclooxygenases 1 and 2

The inhibition of cyclooxygenases 1 (COX-1) and 2 (COX-2) was described in detail by Strugała et al. [67]. This experiment, established on the basis of a modified method given in the work by Jang and Pezzuto [68], was conducted by a spectrophotometric measurement of inhibition of the cyclooxygenases’ activity COX-1 and COX-2. In short, the experimental procedure was as follows: to a cuvette containing Tris-HCl buffer (pH 8.0) the following were successively added: Q 7-MeGlu/Q, hematin (0.1026 mM) and cyclooxygenases (COX-1 or COX-2) at 1 mg/mL. After mixing and incubating (approx. 3 min), TMPD was added at 24.35 mM. To initiate the reaction, arachidonic acid was added at a concentration of 35 mM. The final volume of the sample was 1 mL. The concentrations of studied compounds fluctuated in the range 20–100 µM for Q 7-MeGlu and 5–50 µM for Q. Absorbance of the sample was followed for 3 min by measuring it at 1 min intervals, using a spectrophotometer at a wavelength of 611 nm (Cary 100 Bio, Varian) in relation to a reference sample. The measurements were carried out at room temperature. The control sample contained the right amount of solvent (methanol) instead of the studied compounds. The plot of the relation between the degree of enzymes inhibition versus Q 7-MeGlu or Q concentration was the basis to determine the IC_50_ parameter (linear equations: *R*^2^ = 0.9867 and *R*^2^ = 0.9903, respectively). The experiment was performed in five replicates (*n* = 5).

#### 3.6.6. Binding to Human Serum Albumin

Analysis of Q 7-MeGlu and Q interaction with human serum albumin (HSA) was performed according to the procedure described by Strugała et al. [46]. The fluorescence measurements were performed on a Cary Eclipse fluorimeter equipped with 1.0 cm quartz cells and a thermostat bath. All quenching experiments were performed at 300, 305, 310 and 315 K for HSA in a phosphate buffer solution of pH 7.4 and final concentration 1.5 × 10^−5^ M. The excitation wavelength was set at 280 nm (excitation of the Trp and Tyr), and the emission spectra were read at 285–460 nm. The excitation and emission slits were both set to 5 nm. Our method consisted in tracking the quenching of natural HSA fluorescence caused by Q 7-MeGlu and Q (of final concentrations 1, 2, 4, 6, 8, 10, 12, 14 and 16 µM) added successively. The stock solutions of Q 7-MeGlu and Q were prepared by dissolving in methanol. Control sample contained appropriate amount of methanol. The final amount of methanol was always less than 2%, and it had been verified that such amounts of the solvent do not affect the fluorescence of HSA. The experiment was performed in three independent replicates (*n* = 3).

#### 3.6.7. Statistical Analysis

Data are shown as mean values ± standard deviation (SD). The results were analyzed by one-way ANOVA followed by Duncan’s test. *p* values < 0.05 were considered statistically significant. The program Statistica 12.0 (StatSoft, Kraków, Poland) was used for all statistical calculations.

## 4. Conclusions

Quercetin is the most widely distributed flavonol in plant foods, which shows a broad spectrum of beneficial biological properties. However, due to its low water solubility, orally administered quercetin is poorly absorbed and, therefore, its therapeutic potential is limited. The presence of a large polar moiety, e.g., a sugar, bound to a flavonoid molecule increases the water solubility and may result in better absorption and bioavailability. Therefore, research on the biological activity of flavonoid glycosides plays a vital role in understanding the mechanisms of their absorption and actions in the body. A powerful tool for regioselective flavonoid glycosylation are biotransformations produced by fungi. From our previous studies it followed that *B. bassiana* is able to catalyze the regioselective reaction of glycosylation on numerous flavonoids by attaching the 4-*O*-methylated derivative of glucopyranose in lieu of glucose. Inspired by the broad spectrum biological activity of quercetin, we obtained the derivative quercetin 7-*O*-β-d-(4″-*O*-methyl)glucopyranoside (Q 7-MeGlu) by means of biotransformation conducted by *B. bassiana* AM278, and tested its activity in biological assays compared to that of quercetin (Q). To the best of our knowledge, the biological activity of Q 7-MeGlu has not been the matter of scientific analyses until now. Results of the study showed that the attached 4-*O*-methylated derivative of glucopyranose moiety retained its biological activity in vitro. The results of fluorometric studies and ^1^H-NMR techniques proved that Q7-MeGlu is able to interact not only with the membrane surface by its interference with phospholipid polar heads, but also with upper regions of hydrophobic acyl chains of phospholipids, simultaneously causing a rigidifying effect in those areas. Q 7-MeGlu showed slightly lower antioxidant activity compared to Q (IC_50_^Q 7-MeGlu^ = 5.47; IC_50_^Q^ = 4.49 μM, respectively) and effectively protected lipid membranes against peroxidation. The analyzed antioxidant activity in the phosphatidylcholine liposome membrane can be explained in the following way: Q 7-MeGlu when embedded in the superficial polar region is also able to interfere with the hydrophobic regions of lipid bilayers, reducing the free radicals concentration in the proximity of the membrane and playing a role as a kind of barrier against free radicals which are able to penetrate into the surface of the membrane. Membrane rigidification caused by Q 7-MeGlu is likely to be responsible for the suppression of lipid peroxidation by hindering the diffusion of free radicals into the membrane and by decreasing their reaction efficiencies. The studies have also shown that Q 7-MeGlu and Q inhibit activity of the anti-inflammatory enzymes COX-1 (IC_50_^Q 7-MeGlu^ = 45.06; IC_50_^Q^ = 15.72 μM,) and COX-2 (IC_50_^Q 7-MeGlu^ = 49.23; IC_50_^Q^ = 16.96 μM). The studies provided us with a framework for elucidating the Q 7-MeGlu-HSA binding mechanisms (Q 7-MeGlu < Q). The process of binding Q 7-MeGlu and Q with human serum albumin was a spontaneous molecular interaction connected with hydrogen bonds and van der Waals forces which played a major role in the interaction.

## Figures and Tables

**Table 1:** Quenching changes in fluorescence intensity (for MC540 probe) and anisotropy (for TMA-DPH and DPH probes) in the presence of quercetin 7-*O*-β-d-(4″-*O*-methyl)glucopyranoside (Q 7-MeGlu) and quercetin (Q) used at concentration range 1–10 µM in phosphatidylcholine liposomes (PC). Negative values indicate a decrease in fluorescence intensity as compared to control, while positive values indicate an increase in fluorescence intensity/anisotropy as compared to control. Means labelled with asterisk (*) are significantly different (*p* < 0.05) from control.

Compound/Concentration (µM)	MC540 Intensity Change from Control	TMA-DPH Anisotropy Change from Control	DPH Anisotropy Change from Control
**Q 7-MeGlu**			
1	−0.0269 ± 0.0137 *	0.0518 ± 0.0144 *	0.0614 ± 0.0359 *
5	−0.0955 ± 0.0010 *	0.0845 ± 0.0218 *	0.1257 ± 0.0193 *
10	−0.1240 ± 0.0189 *	0.1005 ± 0.0328 *	0.2674 ± 0.0584 *
**Quercetin**			
1	−0.2890 ± 0.0289 *	0.1619 ± 0.0133 *	0.2943 ± 0.0708 *
5	−0.4585 ± 0.0660 *	0.2449 ± 0.0122 *	0.7464 ± 0.0649 *
10	−0.5580 ± 0.0012 *	0.3708 ± 0.0674 *	0.9661 ± 0.0511 *

**Table 2:** Parameters of ^1^H-NMR spectra at 325 K of DPPC liposomes and DPPC liposomes with addition of quercetin 7-*O*-β-d-(4″-*O*-methyl)glucopyranoside (Q 7-MeGlu) and quercetin (Q) at concentration 0.24 mM (ratio DPPC to Q 7-MeGlu or Q was 150:1, *v*/*v*).

Liposome Composition	Parameter					
	ν -N^+^-(CH_3_)_3out_ (ppm)	ν -N^+^-(CH_3_)_3in_ (ppm)	Δδ (ppm)	ν -CH_2_ (ppm)	ν -CH_3_ (ppm)	I_out_/I_in_
**DPPC**	0.0365	0.0313	0.2280	0.1121	0.0633	0.9977
**DPPC + Q 7-MeGlu**	0.0882	0.0323	0.1000	0.1179	0.0627	0.8646
**DPPC + Quercetin**	0.0799	0.0261	0.1560	0.1181	0.0604	1.3305

**Table 3:** Compilation of IC_50_ in the process of inhibition of AAPH induced oxidation of phosphatidylcholine liposomes and inhibition of COX-1 and COX-2 enzyme activity by quercetin 7-*O*-β-d-(4″-*O*-methyl) glucopyranoside (Q 7-MeGlu) and quercetin. Means labeled with an asterisk (*) are significant (*p* < 0.05) in the same row.

Compound		IC_50_ (µM)	
	AAPH ^PC^	COX-1	COX-2
**Q 7-MeGlu**	5.47 ± 0.47 *	45.06 ± 2.51 *	49.23 ± 5.55 *
**Quercetin**	4.49 ± 0.24 *	15.72 ± 1.73 *	16.96 ± 2.43 *
**L(+) ascorbic acid**	129.46 ± 12.44 *^1^	-	-
**Indomethacin**	-	25.57 ± 0.64 *^2^	21.25 ± 1.90 *^2^

^1^ Strugała et al. [46]; ^2^ Strugała et al. [47].

**Table 4:** Quenching (*K_sv_*) and binding (*K_b_*) constants and thermodynamic parameters (*n*, Δ*G*, Δ*H* and Δ*S*) of quercetin 7-*O*-β-d-(4″-*O*-methyl)glucopyranoside (Q 7-MeGlu) and quercetin and human serum albumin at different temperatures. Means labelled with asterisk (*) are significant (*p* < 0.05).

Compound	*T(K)*	*K_sv_* (·10^4^ M^−1^)	*K_b_* (·10^4^ M^−1^)	*n*	∆G (kJ/M)	∆*H* (kJ/M)	∆S (J/(M·K))
**Q 7-MeGlu**	300	3.837 ± 0.192 *	9.355 ± 1.442 *	1.082 ± 0.025	−12.380 ± 0.286	−164.46 ± 5.590	−504.768 ± 18.919
	305	3.545 ± 0.025 *	5.043 ± 1.095 *	1.024 ± 0.055	−11.810 ± 0.724		
	310	3.412 ± 0.045 *	0.071 ± 0.021 *	0.634 ± 0.026 *	−7.323 ± 0.343 *		
	315	3.182 ± 0.013 *	0.009 ± 0.001 *	0.437 ± 0.011 *	−5.196 ± 0.151 *		
**Quercetin**	300	17.510 ± 0.152 *	88.369 ± 5.236 *	1.048 ± 0.145	−13.444 ± 1.962	−132.616 ± 3.859	−390.267 ± 12.102
	305	16.719 ± 0.135 *	69.072 ± 2.726 *	1.014 ± 0.176	−13.167 ± 2.319		
	310	15.632 ± 0.109 *	2.486 ± 0.421 *	0.801 ± 0.055	−10.755 ± 0.812 *		
	315	13.718 ± 0.058 *	0.345 ± 0.091 *	0.645 ± 0.023 *	−0.899 ± 0.377 *		

## Supplementary Materials

Supplementary materials are available online.
