# Supplementary material for: Bioactivity In Vitro of Quercetin Glycoside Obtained in Beauveria bassiana Culture and Its Interaction with Liposome Membranes
Source: Molecules. 2017 Sep 11;22(9):1520. doi: 10.3390/molecules22091520 (PMC6151435; doi:10.3390/molecules22091520)
Supplement: Supplementary file 1 [file molecules-22-01520-s001.pdf]

SUPPLEMENTARY MATERIALS

# Bioactivity *in vitro* of quercetin glycoside obtained in *Beauveria bassiana* culture and its interaction with liposome membranes

Paulina Strugała *[1], Tomasz Tronina [2], Ewa Huszcza [2] and Janina Gabrielska [1]

[1] Department of Physics and Biophysics, Wrocław University of Environmental and Life Sciences, Norwida 25, 50-375 Wrocław, Poland.
[2] Department of Chemistry, Wrocław University of Environmental and Life Sciences, Norwida 25, 50-375 Wrocław, Poland.
*Corresponding Author: E-mail address: paulina.strugala@upwr.edu.pl; tel: +48 713205167

**Table of Contents:** **Page**

UV spectra of quercetin (**Q**) and guercetin 7-O-β-D-(4''-O-methyl)glucopyranoside (**Q 7-MeGlu**) S2
HR ESI-MS spectrum of guercetin 7-O-β-D-(4''-O-methyl)glucopyranoside (**Q 7-MeGlu**) S2
$^{1}$H-NMR and $^{13}$C-NMR spectra of quercetin (**Q**) S3
$^{1}$H-NMR and $^{13}$C-NMR spectra of quercetin 7-O-β-D-(4''-O-methyl)glucopyranoside (**Q 7-MeGlu**) S4
$^{13}$C-NMR fragments of the spectra of quercetin (**Q**) and quercetin 7-O-β-D-(4''-O-methyl)glucopyranoside (**Q 7-MeGlu**) S5
$^{1}$H -$^{1}$H NMR (COSY) spectrum of quercetin 7-O-β-D-(4''-O-methyl)glucopyranoside (**Q 7-MeGlu**) S6
$^{1}$H -$^{1}$H NMR (COSY) spectrum of quercetin 7-O-β-D-(4''-O-methyl)glucopyranoside (**Q 7-MeGlu**) S6

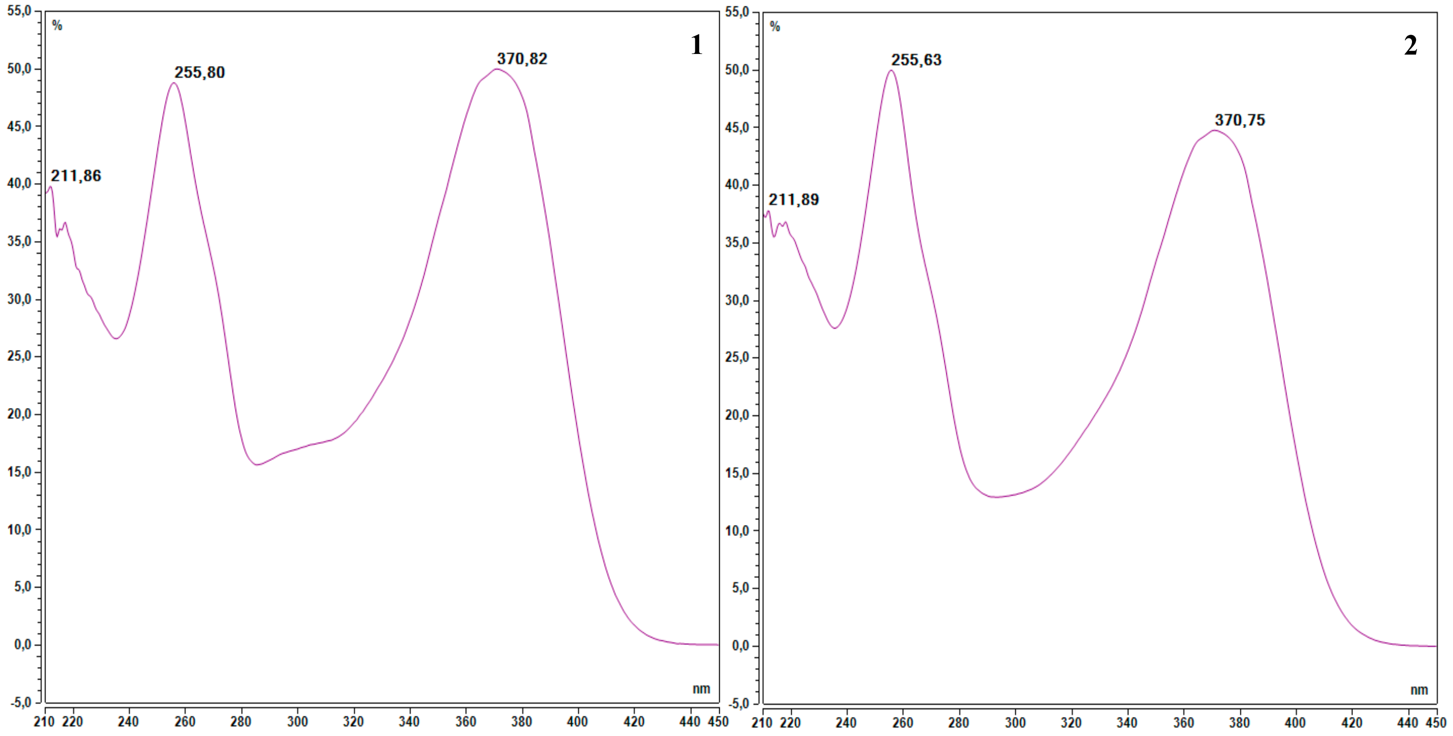


**Figure S1**. UV spectra of quercetin (**Q**) and guercetin 7-O-β-D-(4''-O-methyl)glucopyranoside (**Q 7-MeGlu**),(MeOH, Temp 20°C)

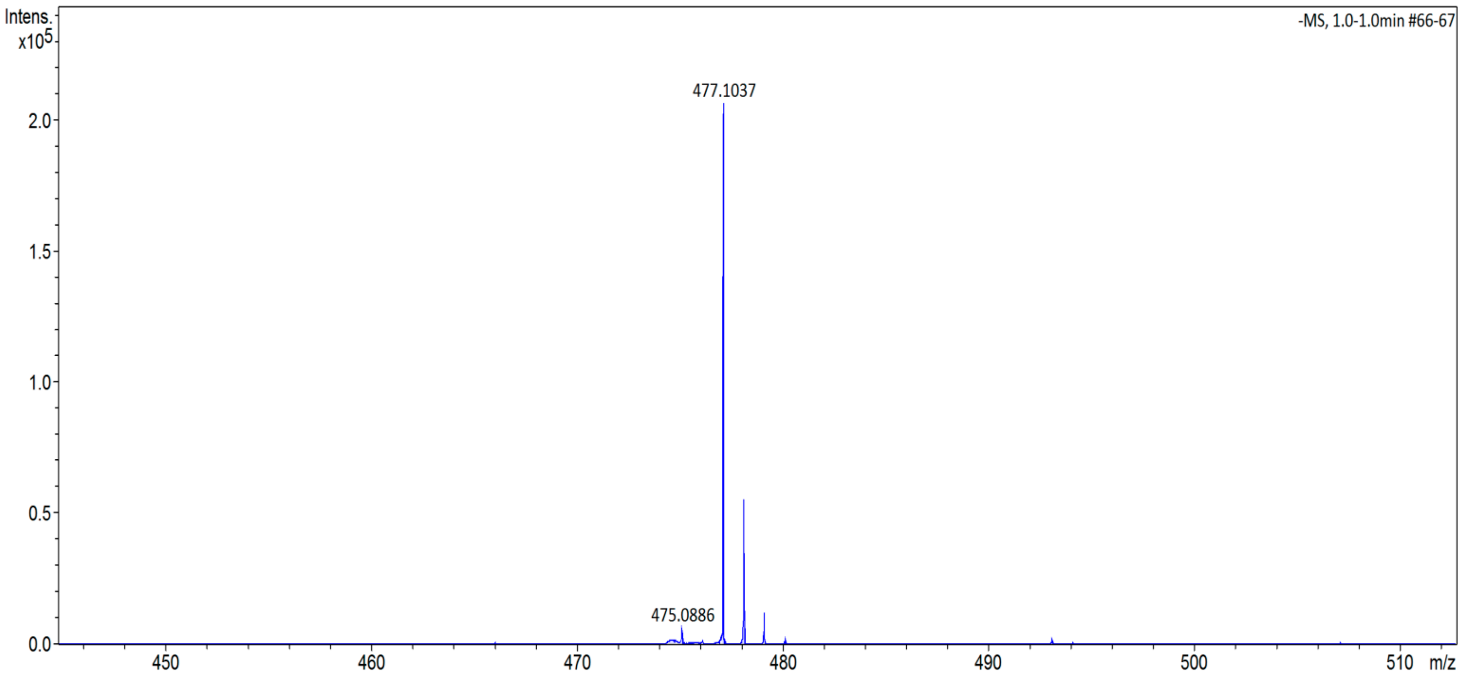


**Figure S2**. HR ESI-MS spectrum of guercetin 7-O-β-D-(4''-O-methyl)glucopyranoside (**Q 7-MeGlu**)

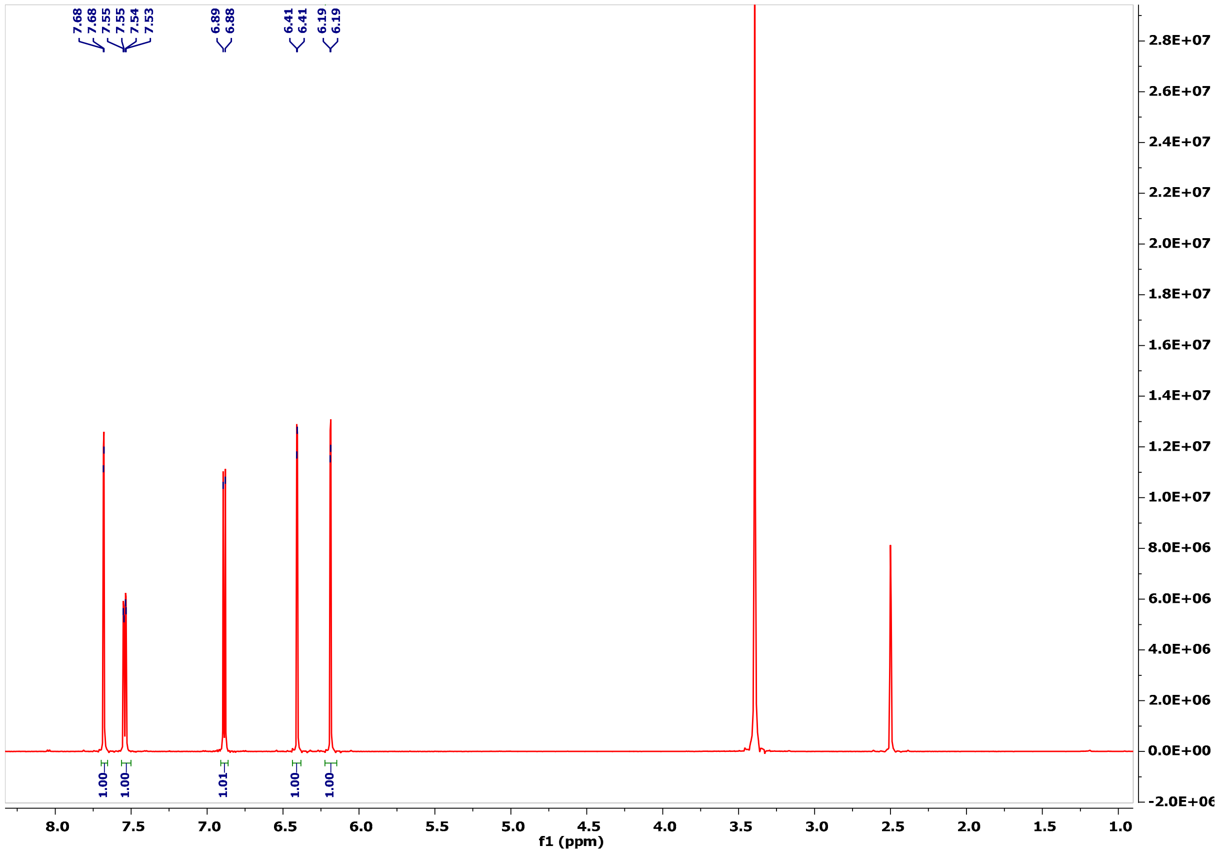


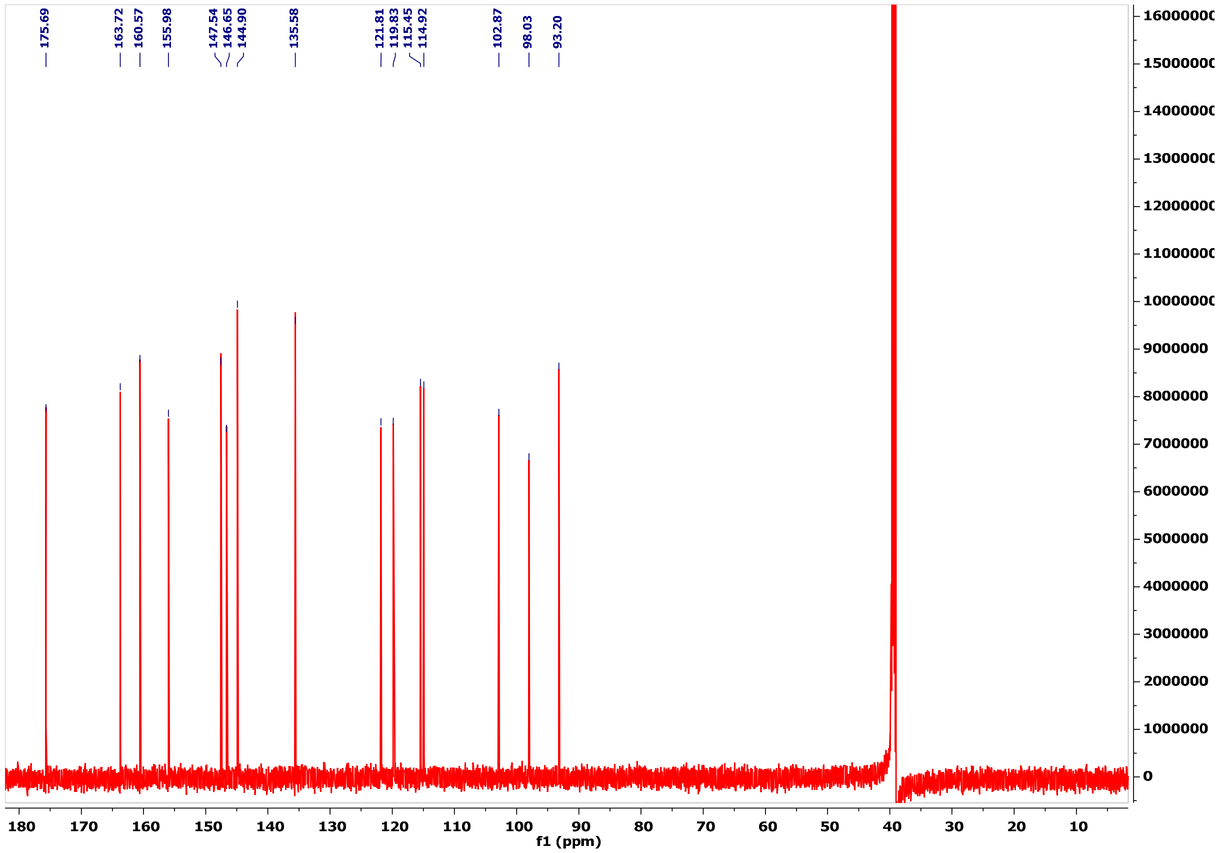


**Figure S3**. $^{1}$H-NMR (Top) and $^{13}$C-NMR (Bottom) spectra of quercetin (Q)(600 and 151 MHz, DMSO-d$_6$, Temp. 28 °C)

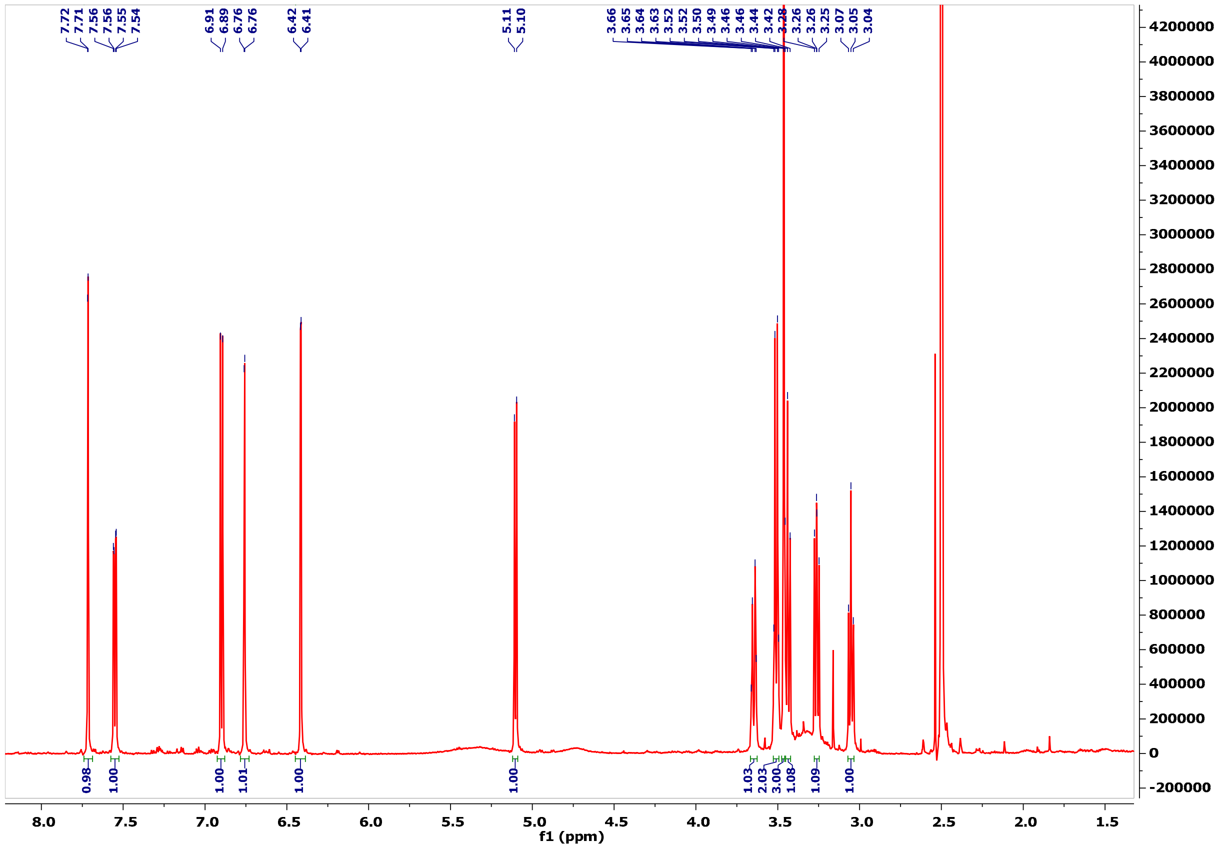


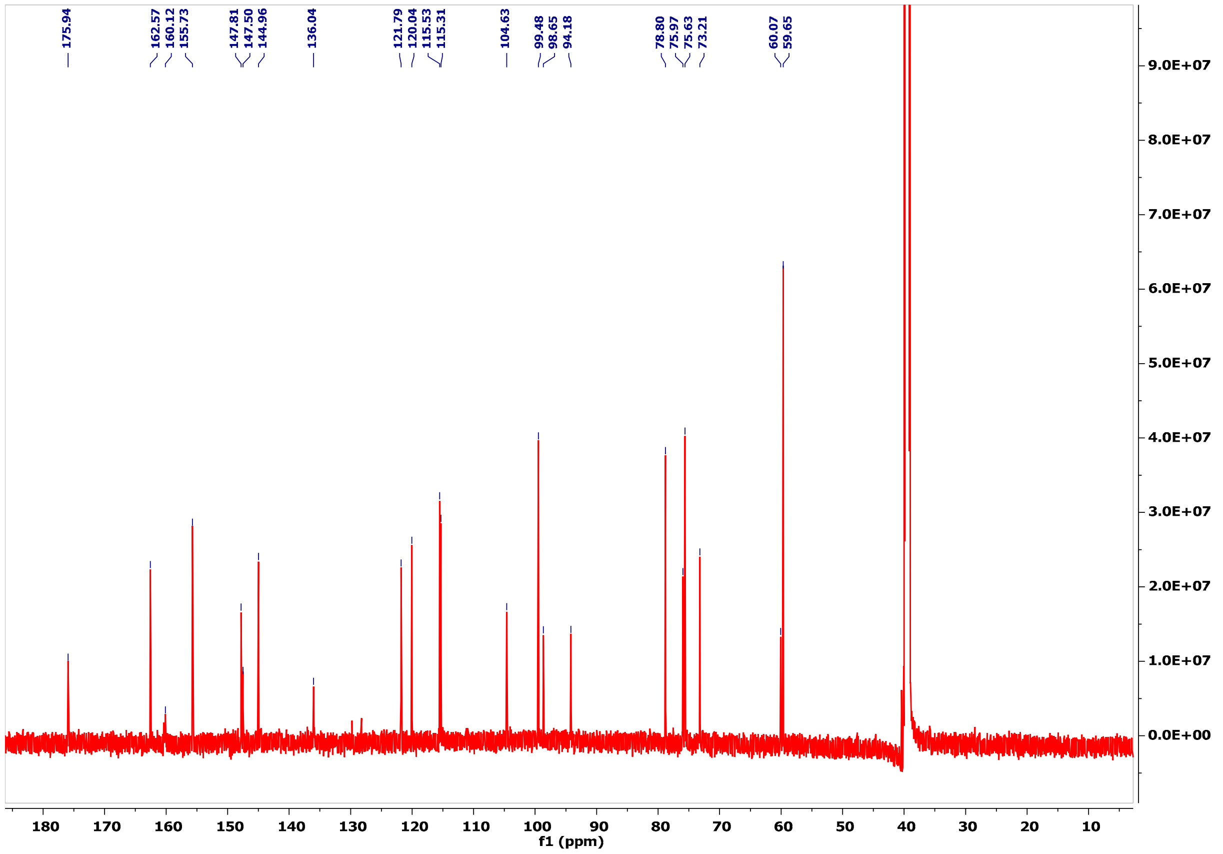


**Figure S4.** $^{1}$H-NMR (Top) and $^{13}$C-NMR (Bottom) spectra of quercetin 7-O-β-D-(4''-O-methyl)glucopyranoside (**Q 7-MeGlu**) (600 and 151 MHz, DMSO-$d_6$, Temp. 28 °C)

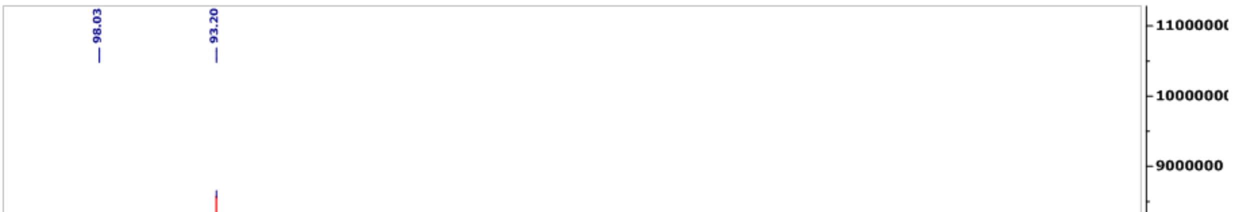

**Figure S5.** $^{13}$C-NMR fragments of spectra of quercetin (**Q**) (Top) and quercetin 7-O-β-D-(4''-O-methyl)glucopyranoside (**Q 7-MeGlu**) (Bottom)( 151 MHz, DMSO-d$_6$, Temp. 28 °C)

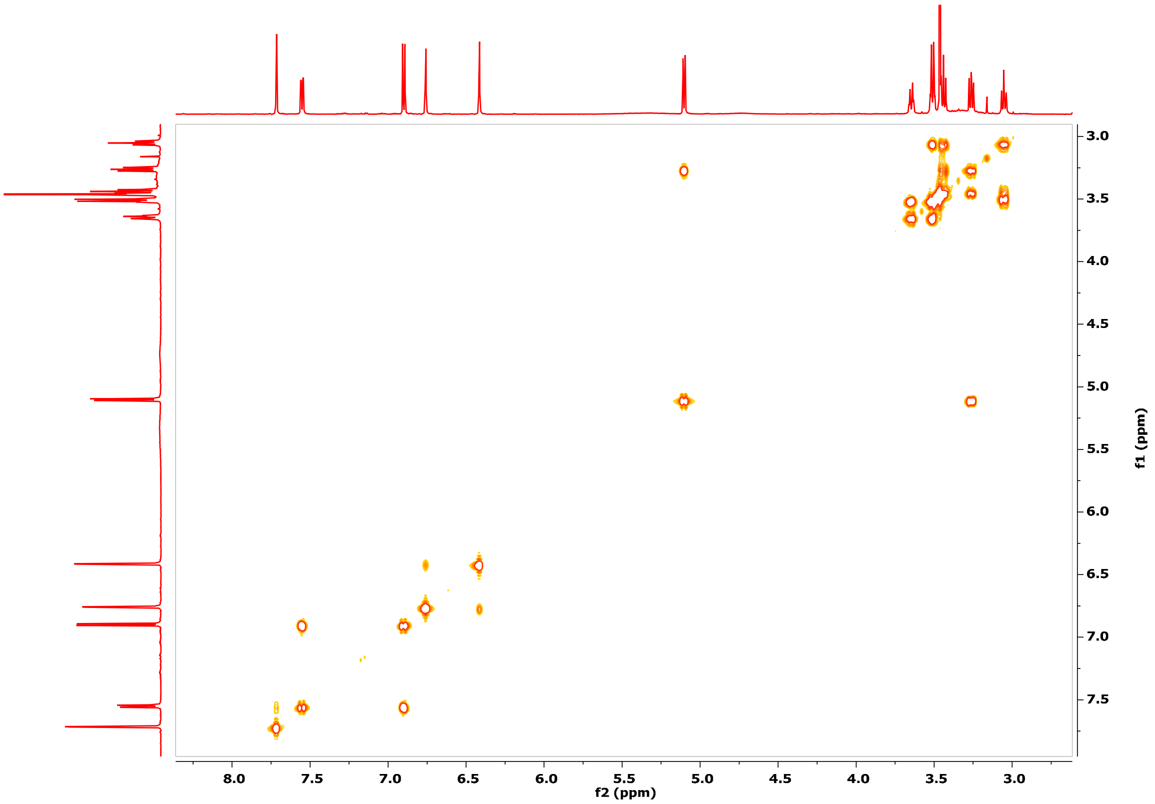


**Figure S6**. $^1$H -$^1$H-NMR (COSY) spectrum of quercetin 7-O-β-D-(4''-O-methyl)glucopyranoside (**Q 7-MeGlu**) (600 /600 MHz, DMSO-d$_6$, Temp. 28 °C)

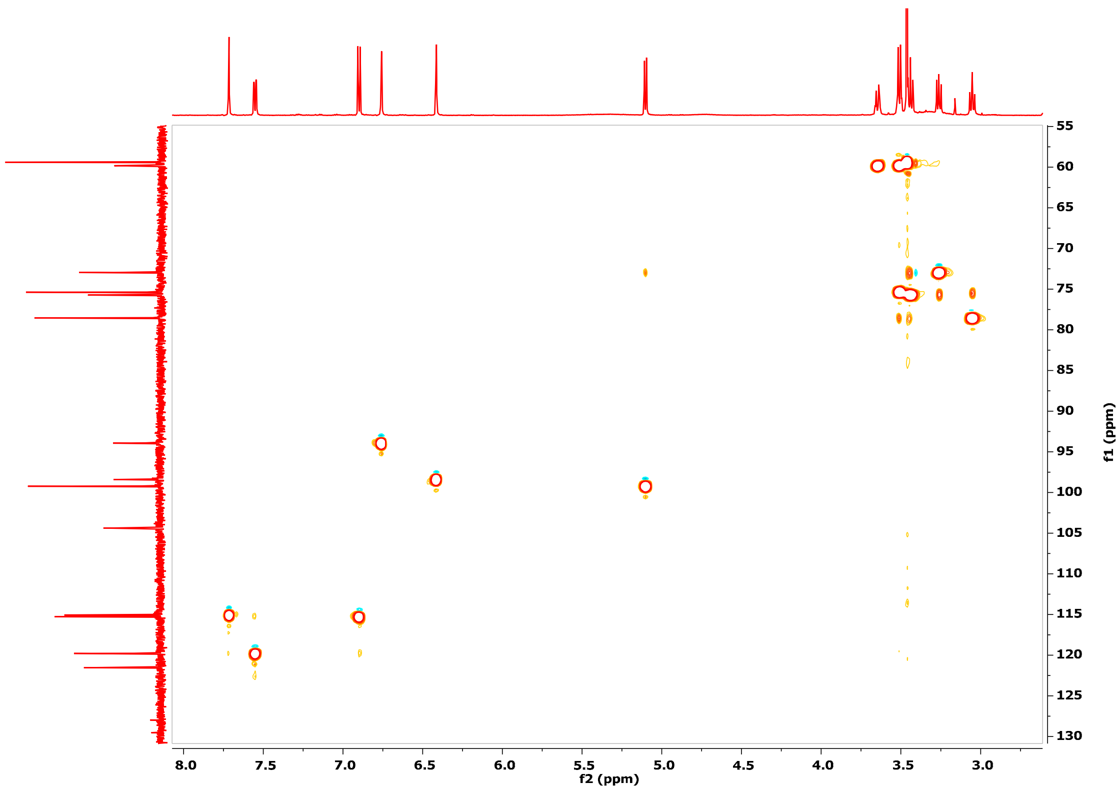


**Figure S7**. $^{1}H$ -$^{13}C$-NMR (HSQC) spectrum of quercetin 7-O-β-D-(4''-O-methyl)glucopyranoside (**Q 7-MeGlu**) (600 /151 MHz, DMSO-$d_6$, Temp. 28 °C)
